# China’s carbon emissions structure and reduction potential on the supply-side and demand-side of energy: Under the background of four influencing factors

**DOI:** 10.1371/journal.pone.0255387

**Published:** 2021-08-06

**Authors:** Xinwen Wan, Tangyang Jiang, Shuangqi Li, Jun Nie

**Affiliations:** 1 School of Internet, Anhui University, Anhui, China; 2 School of Finance, Chongqing Technology and Business University, Chongqing, China; 3 School of Public Affairs, Chongqing University, Chongqing, China; Institute for Advanced Sustainability Studies, GERMANY

## Abstract

In recent years, the issues related to carbon emissions and environment have attracted extensive attentions. Considering four scenarios (the energy conversion, energy capital savings and loans, energy exports and cement production carbon emissions), this paper adopts the energy consumption method and input-output method to analyze China’s carbon emissions structure on the supply-side and demand-side of energy, and finally provides policy recommendations for China’s structural emission reduction. The results show that, if the four influencing factors were not considered, the measurement of carbon emissions from the final demand was 44.91% higher than the baseline scenario, 12.36% lower than the baseline scenario from intermediate demand, and 10.23% lower than the baseline scenario from the total. For China’s carbon emissions structure on the supply-side of energy, the carbon emissions from high-carbon energy, represented by raw coal, accounted for 66.805% of the total energy-related carbon emissions, while the carbon emissions from low-carbon energy, represented by natural gas, only accounted for 2.485%. For China’s carbon emissions structure on the demand-side of energy, the carbon emissions from intermediate demand (enterprise production) accounted for more than 95% of total energy-related carbon emissions, while the carbon emissions from final demand (residents and government use) accounted for less than 5%. For each specific industry in intermediate demand for energy, the heavy industry, electric power, fossil energy, and chemical industry have high carbon emissions and low carbon emissions efficiency. However, the agriculture, construction, light industry, and service are the opposite. Finally, we provide policy recommendations for improving the accuracy of carbon emissions measurement and carbon emissions efficiency.

## Introduction

At present, climate change has become an environmental issue of general concern in society [1].The greenhouse effect caused by excessive carbon emissions is blamed for climate change [2]. According to the latest BP Statistical Review of World Energy, in 2019, China’s carbon emissions ranked first in the world, reaching 9825.8 million tons, accounting for 28.76% of the total global carbon emissions. Therefore, controlling carbon emissions is the top priority to solve environmental problem. In this context, the measurement of carbon emissions and its influencing factors analysis become significantly important as it is the cornerstone for carbon emissions related research.

From the sources, energy consumption and biomass decomposition are the main producers of carbon emissions [3, 4]. Many scholars have widely concerned the dominant factors affecting the measurement of carbon emissions. However, some implicit factors that affect the measurement of carbon emissions cannot be ignored. For example, energy conversion, energy capital deposits and loans and energy exports do not produce carbon emissions in these processes. In addition, the cement production processes involves implicit carbon emissions of energy consumption [5].

Under the background of sustainable development and green development, energy conservation and carbon emissions reduction have become important tasks for China. At the beginning of the 21st century, the Chinese government has launched a series of low-carbon development policy measures [6]. However, as a developing country, China has been developing at high speed for more than a decade, which leads to continued growth in energy consumption [7]. Naturally, the implementation of emissions reduction tasks has always faced enormous challenges. With the increasingly serious environmental problems, the Chinese government is making more efforts to protect the environment. How to measure carbon emissions more accurately is particularly critical.

The energy consumption, life cycle assessment, and input-output method are three mainstream methods for measuring the carbon emissions. Each method has its advantages and disadvantages. The energy consumption method refers to carbon emissions caused by energy combustion based on statistical data, energy consumption, and carbon emissions coefficient [8, 9]. The advantage of the energy consumption method is that the data required for analysis is more flexible and accurate [10]; the shortcoming of the energy consumption method is that other non-energy consumption carbon emissions, such as carbon emissions from cement processes, are easy to be ignored. The life cycle assessment method is usually based on activity links, requiring detailed study of waste energy emissions from energy demand, raw material utilization, and activities during the life of the measurement target to measure the carbon emissions [11, 12]. Although this method can be specific to the raw material mining, transportation, manufacturing and other aspects of the product [13, 14], the life cycle assessment method also has shortcomings such as large computational workload. The input-output method measures the carbon emissions by the input-output table in which a series of internal departments are arranged in a certain period [15]. The advantage of the input-output method is that the direct and indirect carbon emissions can be estimated by the direct consumption coefficient of the product and the complete consumption coefficient [16]; the deficiency is that the measurement results are not as accurate as the above-mentioned two methods.

To include four influencing factors (the energy conversion, energy capital savings and loans, energy exports and cement production), this paper comprehensively considered the advantages and disadvantages of each method, adopts the energy consumption method and input-output method to analyze the carbon emissions structure from the energy consumption on the supply-side and demand-side of energy, as well as carbon efficiency from different sectors. Carbon emissions from the energy system and the industry system are unified by input-output analytical framework, which provides policy recommendations for China’s structural emission reduction.

The remainder of this paper is as follows. The second part is literature review; the third part is model construction and data processing; the fourth part is the result and analysis; the fifth part is the conclusion and policy recommendations.

## Literature review

Regarding analysis about energy-related carbon emissions, the existing literature mainly took energy structure or a certain industry as the starting point of research. The analysis about carbon emissions from energy structure mainly studied the impact of energy consumption on the growth of carbon emissions in the energy system, while the analysis about carbon emissions from a certain industry mainly studied the impact of energy consumption on carbon emissions in a certain industry. However, these researches did not involve the entire energy system and industrial system. The results cannot fully reflect the carbon emissions structure on the supply-side and demand-side of energy. Next, we sorted literatures about carbon emissions from the energy structure or a certain industry, and carbon emissions efficiency. These works provide a theoretical basis for our research.

### Carbon emissions from the energy structure or a certain industry

Many scholars studied carbon emissions from the perspective of the energy structure. For instance, Hua (2011) [17] studied the relationship between carbon emissions and energy structure, and found that the peak of energy consumption would lag behind the peak of carbon emissions with the turning point of the world energy structure coming in 2020–2025. Zhu et al. (2015) [18] studied China’s evolution of energy structure in unrestricted free carbon emissions and carbon tax-based policies. Many scholars also studied carbon emissions from the perspective of a certain industry. However, few scholars analyzed the carbon emissions structures on the supply-side and demand-side of energy considering their influencing factors [19–21]. Since electricity, cement and heavy industries are high energy consumption industries, most studies about carbon emissions concentrated on these industries [22–24]. In terms of other industries, Tang et al. (2017) [25] proposed a factor decomposition model for analyzing the carbon emissions of energy consumption in the tourism industry from the perspective of the tourism region life cycle. Lin and Lei (2015) [26] evaluated the carbon emissions of energy consumption in the Chinese food industry based on the LMDI (Logarithmic Mean Divisia Index, LMDI) method.

By summarizing the above literature, we can conclude that most of existing literatures about carbon emissions concentrated in a specific industry. Few literatures systematically analyzed the carbon emissions situation and the carbon emissions structure characteristics of all industries on the demand-side of energy. Fewer literatures develop such a study on the basis of considering the four factors (the energy conversion, energy capital savings and loans, energy exports and cement production carbon emissions). However, this study is conducive to more accurately depicting the carbon emissions on the supply-side and demand-side of energy, and provides theoretical analysis basis and practical guidance for the Chinese government to control carbon emissions more accurately from the supply-side and demand-side of energy.

### Carbon emissions efficiency

Many scholars used different models to study carbon emissions efficiency from different perspectives. For example, Dong et al. (2014) [27] used a three-stage DEA (Data Envelopment Analysis, DEA) model to estimate regional carbon emissions efficiency in China. Based on Supper Efficiency DEA model, Liu and Hu (2015) [28] investigated the carbon emissions efficiency of 30 provinces (including cities and districts) in China from 1997 to 2011, and applied SFA (Stochastic Frontier Approach, SFA) to eliminate the influence from the environment and random error factors. Zhang et al. (2016) [29] studied the carbon emissions efficiency and energy efficiency of the industrial sector, which is of great significance to the government’s formulation of regional industrial policies. Wu et al. (2016) [30] comprehensively assessed the steel industry network and its environmental performance in energy and carbon emissions, noting that by adopting IS (Industrial Symbiosis, IS) measures, overall efficiency and energy efficiency can be improved. Based on the SBM (Slack Based Model, SBM), Liu et al (2016) [31] analyzed the data of 30 regions in China from 2000 to 2011, and quantitatively studied the current situation of CO2 emission efficiency in various provinces, the characteristics of energy consumption structures in different provinces, and the differences between provinces. The results provided theoretical support for guiding energy consumption, controlling environmental pollution, and promoting healthy economic development. Meng et al. (2016) [32] used the DEA to evaluate the regional energy efficiency and carbon emissions efficiency in China.

By analyzing the above literatures, we can draw the conclusion that most of previous studies about carbon emissions efficiency focused on the provincial or regional level. Few literatures analyzed the carbon emissions efficiency from the industrial level. Meanwhile, the four factors in this paper will affect the calculation of carbon emissions of various industries on the demand-side of energy. Few literatures about the carbon emissions efficiency of industries rarely consider these four factors. However, this study is conducive to more accurately depicting the carbon emissions status of energy demand side industries, and provides theoretical analysis basis and practical guidance for the Chinese government to carry out the optimization of carbon emissions efficiency from the industrial level.

### The academic contribution of this paper

Compared with the existing literatures, the three contribution points reflected in this as follows. First, this paper compared measurement error of carbon emissions with and without considering four influencing factors (energy conversion, energy capital deposits, energy exports, and cement production processes). Second, by the energy consumption method and input-output method, this paper analyzed carbon emissions structure in energy consumption structure in the entire energy system and industrial system from the perspective of the supply-side and demand-side of energy considering the above four influencing factors. Third, from the perspective of carbon emissions efficiency, carbon emissions efficiency of various industries was analyzed. These contributions in this paper provide theoretical and empirical evidence for the energy structure optimization, energy saving and carbon emissions reduction from the supply-side and demand-side of energy.

## Theoretical basis

This part mainly includes two aspects. One is the theory of energy consumption method, the other is the theory of input-output analysis.

### Energy consumption method

In the process of national production, energy as an intermediate input participates in national economic activities. However, it should be noted that in the process of energy input participating in national production, carbon emissions from energy combustion are unexpected outputs [33]. The energy consumption method to measure carbon emissions is based on statistical data such as the energy consumption and carbon emissions coefficient of each energy source. The data selection of this method is more flexible, and the suitable data can be selected for analysis according to specific problems. Therefore, many scholars use this method to measure carbon emissions [31–34]. According to the consumption data of China’s final demand and the carbon emissions coefficient of energy released by IPCC (Intergovernmental Panel on Climate Change, IPCC), this paper adopts the energy consumption method to measure the carbon emissions on the supply-side of energy, and further analyzes the structural characteristics of carbon emissions.

### Input-output analysis

Input-output analysis is a method for studying the balanced relationship among various sectors of the national economy. Based on the assumption of general equilibrium, the dependence of product quantity of each department is expressed as a system of equations. Then, according to the statistical materials, a matrix or chessboard balance table is made to show the overall picture of the balance between supply and demand of products in various sectors of the national economy. From this, the ratio of the total amount of products of each department to that of other departments needed to produce this total amount can be obtained, and the values of relevant parameters in the above equations can be determined [35, 36]. The input-output analysis is mainly based on the input-output table. The direct and indirect carbon emissions can be estimated according to the direct consumption coefficient and the complete consumption coefficient of products [37]. The direct consumption coefficient refers to the total amount of products or services directly consumed by a certain product department under the unit of total output. The complete consumption coefficient refers to the total amount of products or services of each department directly and indirectly consumed for each unit of final products provided by a certain department. The advantage of this measurement method is that it can estimate the embodied carbon emissions. In addition, for the measurement of multi industry carbon emissions, the direct consumption coefficient matrix and the complete consumption coefficient matrix are used to estimate once, which can reduce the workload of industry classification.

## Model

### The measurement of CO_2_ emissions from energy combustion

The measurement model of carbon emissions in this paper comes from the theoretical model found in Liu et al. (2015) and Jiang et al. (2019) [38, 39]. The total amount of CO_2_ emission from energy combustion is the sum of CO_2_ emission from the combustion of each energy source, which can be computed as follows.

$$E=\sum_{i}C_{i}\times W_{i}$$ (1)

where, *C*_*i*_ is the combustion amount of the energy source *i*, and *W*_*i*_ is the carbon emissions coefficient of energy source *i*. According to the energy balance sheet, the segment of the energy used for combustion includes the final consumption of energy, the consumption for thermal power generation, and the consumption for heating, but excluding the part used as raw materials in the industry. To ensure the accuracy of the results, we subtracted the amount of unburned energy from the total energy consumption. Then *C*_*i*_ can be computed as follows. This model refers to Liu et al. (2015) and Jiang et al. (2019) [38, 39].

$$C_{i}=C_{i}^{T}+C_{i}^{P}+C_{i}^{H}-C_{i}^{M}$$ (2)

where, $C_{i}^{T}$ is the combustion amount of energy source *i* used for final consumption; $C_{i}^{P}$ is the combustion amount of energy source *i* used for thermal power generation; $C_{i}^{H}$ is the combustion amount of energy source *i* used for heating, and $C_{i}^{M}$ is the amount of energy source *i* used for industrial raw materials.

The carbon emissions coefficient of energy combustion is computed as follows.

$$W_{i}=T_{i}\times Q_{i}$$ (3)

where, *T*_*i*_ is the carbon emissions per unit of heat produced from the combustion of energy source *i*; *Q*_*i*_ is the average low calorific value of burning energy source *i*.

The coal mining industry, petroleum and natural gas exploitation industry, petroleum, cooking, and nuclear fuel products industry, and the gas production and supply industry provide the total energy consumption for the 42 industries in China. However, the energies offered by the four energy industries are not all used for the consumption of domestic products. Therefore, whether to eliminate the conversion of energy, the energy fixed capital formation or the energy exports will result in different carbon emissions.

In this study, we discuss the situations where these three parts of energy consumption are subtracted or not. First, without subtracting any of these three parts of energy consumption, the total energy investment for the combustion of all energy industries is as follows.

$$D_{j}=DI_{j}+DF_{j}$$ (4)

where, *D*_*j*_ is the total energy investment for the combustion of energy industry *j*; *DI*_*j*_ is the energy from the intermediate demand for the combustion of energy industry *j*, and *DF*_*j*_ is the energy from the final demand for the combustion of energy industry *j*. If all there three parts are subtracted, then the *D*_*j*_ is as follows.

$$D_{j}=DI_{j}+DF_{j}\text{-}DC_{j}-DE_{j}-DS_{j}$$ (5)

where, *DC*_*j*_ is the energy formed into total fixed capital; *DE*_*j*_ is the energy which has been exported or transferred, and *DS*_*j*_ is the transformational energy generated from energy industry *j*. Then, the carbon emissions coefficient of an energy industry is as follows.

$$e_{j}=\frac{\sum C_{i}\times W_{i}}{D_{j}}$$ (6)

where, *e*_*j*_ is the carbon emissions coefficient of energy industry *j*.

Multiplying the generated energy investment of the energy industry *j* that is used for industry *k* and the coefficient computed in Eq (6), we can measure the carbon emissions $E_{j}^{k}$. The total carbon emissions of industry *k* include the sum of the energy used for industry *k* derived from each energy industry, which can be computed as follows.

$$E^{k}=\sum_{j}E_{j}^{k}=\sum_{j}C_{j}^{k}\times e_{j}$$ (7)

where, *E*^k^ is the carbon emissions of industry *k*; $E_{j}^{k}$ is the energy industry *j* derived carbon emissions that is generated from industry *k*; and $C_{j}^{k}$ is the energy industry *j* generated energy investment that is used for industry *k*.

Based on the above calculation process, from Eqs (2)–(7), we can measure the individual carbon emissions of the 42 industries due to energy combustion. Then we divided the carbon emissions of industry k by the total output of industry *k*.

$$f^{k}=\frac{E^{k}}{T^{k}}$$ (8)

where, *E*^k^ is the carbon emissions of industry *k*, *T*^*k*^ is the output of industry *k*, and *f*^*k*^ is the carbon emissions efficiency of industry *k*. In this paper, we merged 42 sectors into 9 sectors [38]. For details of the merger (the merging process), please see S1 Fig.

### Carbon emissions from cement production

The CO2 generated by cement production accounts for a considerable proportion in China’s total carbon emissions. Therefore, in measuring China’s total carbon emissions, whether to consider the carbon emissions related to cement production can affect the final results. The carbon emissions generated from cement production can be measured as follows.

EC=QC×u (9)

where, EC is the carbon emissions from cement production; QC is the total output of the cement production, and u is the carbon emissions coefficient for cement production. The coefficient used in this study is the coefficient for Portland cement, obtained from Greenhouse Gas Protocol, whose value is 0.5021016.

## Results and analysis

This paper takes China’s carbon emissions in 2012 as the research object. The selected data sources mainly include China Energy Balance Sheet and Input-Output Table for 2012, the average low calorific value of each energy source and the carbon emissions coefficient, the total cement output of the provinces in 2012 and CO_2_ emission factor for cement production processes.

### The measurement of carbon emissions and its influencing factors

In this paper, four influencing factors (the transformational energy, energy invested in gross capital formation, energy exported or transferred, and the emission from the process of cement production) and 16 scenarios were designed. The details of the 16 scenarios are shown in Table 1.

**Table 1:** Scenario design and description.

Scenario	If transformational energy is subtracted (1 is yes, and 0 is no)	If energy invested in the gross capital formation is subtracted (1 is yes, and 0 is no)	If energy is exported (1 is yes, and 0 is no)	If CO_2_ from cement manufacture is added (1 is yes, and 0 is no)
I	0	0	0	0
II	0	0	0	1
III	0	0	1	0
IV	0	0	1	1
V	0	1	0	0
VI	0	1	0	1
VII	0	1	1	0
VIII	0	1	1	1
IX	1	0	0	0
X	1	0	0	1
XI	1	0	1	0
XII	1	0	1	1
XIII	1	1	0	0
XIV	1	1	0	1
XV	1	1	1	0
XVI	1	1	1	1

From a theoretical point of view, we choose the scenario XVI as the baseline scenario (Table 1). Using the above data and method, we can measure the carbon emissions under the scenario XVI and scenario I (Not consider the four factors). The detailed data are shown in Table 2.

**Table 2:** The measurement of carbon emissions under the scenario XVI and scenario I.

Scene Name	CO_2_ from intermediate demand(10^4^t)	CO_2_ from final demand(10^4^t)	Total CO_2_(10^4^t)
I	904367.8	57712.6	962080.4
XVI	1031915.6	39826.2	1071741.8

Note: Due to a large amount of data, only 1 digit after the decimal point was included.

**Fig 1:**
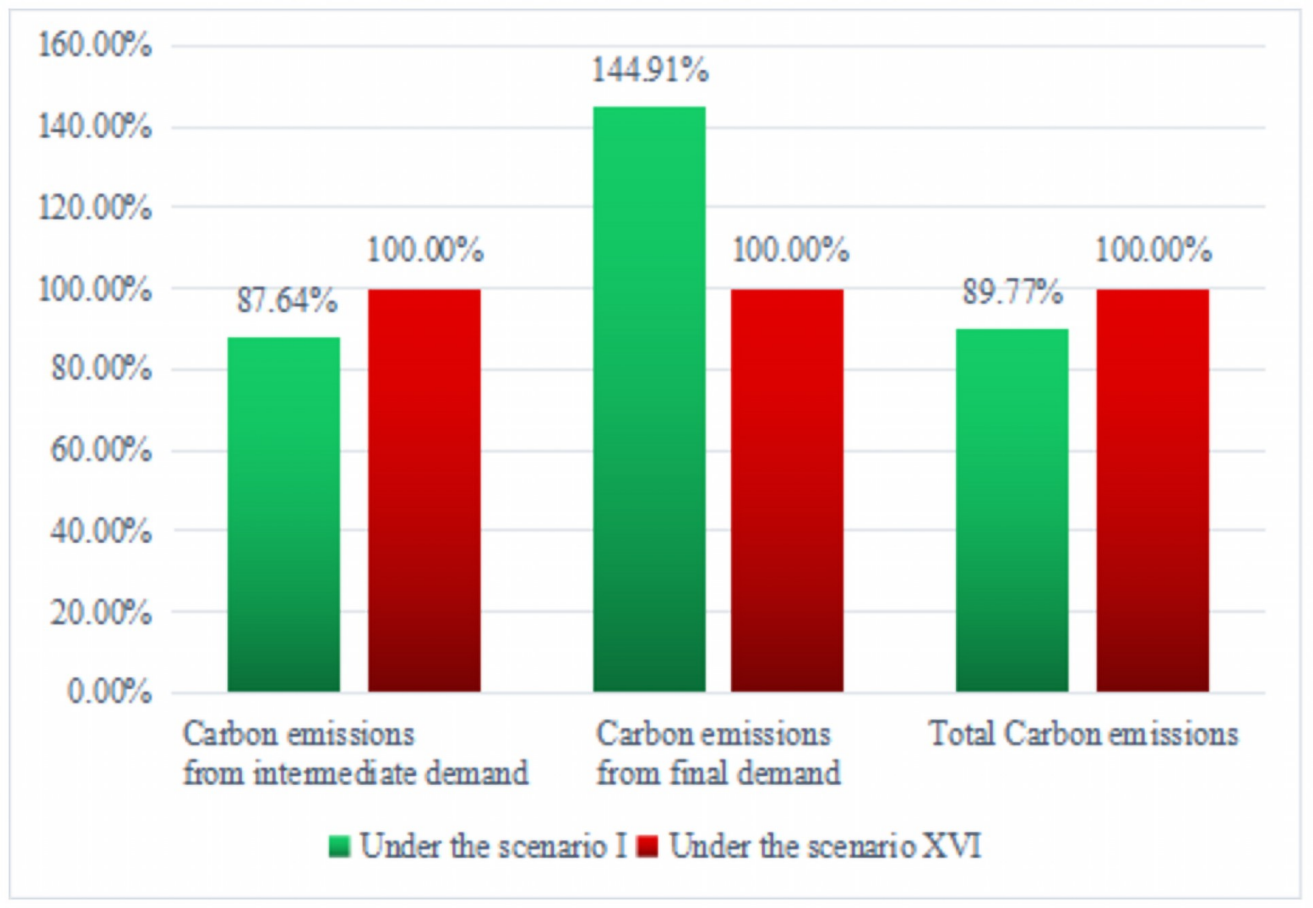
The carbon emissions structure under two scenarios.

As shown in Fig 1, if the four influencing factors were not considered, the measurement of carbon emissions from intermediate demand was 12.36% lower than the baseline scenario, 44.91% higher than the final demand and 10.23% lower than the total.

### Analysis of the carbon emissions structure

#### The carbon emissions structure from energy consumption

According to the above equations, the carbon emissions from burning different energy sources in China for 2012 were measured, and the results are shown in Table 3.

**Table 3:** The CO2 emission from different energy sources.

Categories of energy	CO_2_ emissions (10^4^t)	The share of CO_2_ emissions
Briquettes	852.533316	0.089%
Crude Oil	1513.638333	0.157%
Other Gas	1930.759714	0.201%
Other Coking Products	2566.044624	0.267%
LNG	3807.281008	0.396%
Refinery Gas	3912.853472	0.407%
Converter Gas	5486.499642	0.570%
Coke Oven Gas	5887.496377	0.612%
Kerosene	6059.00322	0.630%
Cleaned Coal	6893.936192	0.717%
Other Petroleum Products	7086.848361	0.737%
LPG	7186.23448	0.747%
Fuel Oil	7454.763682	0.775%
Other Washed Coal	8894.066817	0.924%
Natural Gas	23911.85246	2.485%
Gasoline	24487.71123	2.545%
Blast Furnace Gas	33039.95063	3.434%
Diesel Oil	53534.69814	5.564%
Coke	114854.2229	11.938%
Raw Coal	642719.9995	66.805%

Table 3 can offer two vital sets of information. First, the carbon emissions from burning raw coal continue to contribute more than half (66.805%) of the total amount to China’s energy generation related carbon emissions. This is because the raw coal still accounts for the majority, and accordingly, a large percentage of carbon emissions related to energy production is generated by raw coal combustion in China’s fossil energy consumption structure. Second, for the high-carbon energy sources, such as raw coal, coke, and diesel, the carbon emissions accounts for a large proportion of total carbon emissions; on the contrary, natural gas and liquefied natural gas, which are considered as clean energy sources, generate only a minor amount of carbon emissions. This is not surprising because the natural gas and liquefied natural gas as low-carbon energy sources account for a small proportion of the total consumption in China’s energy consumption structure. On the other hand, raw coal, coke, and diesel and the like as high-carbon energy sources still dominate the energy consumption structure, and the carbon emissions yielded from burning these energy sources are also much higher. In addition, our data suggest that there is a good scope for optimization of China’s energy consumption structure. Therefore, we should try to reduce the proportion of high-carbon energy sources, e.g., coal, and increase the proportion of low-carbon energy sources, e.g., natural gas and liquefied natural gas. Comparing the results of this study with those of Jiang et al. (2019) and Kang & Yang (2020) [39, 40], it is found that the results of the two are basically consistent, which indirectly enhances the reliability of the results of this study.

#### The carbon emissions structure on the supply-side of energy

Using the above data and method, we can measure the carbon emissions from the energy consumption and the carbon emissions from the cement production. The detailed data are shown in Table 4.

**Table 4:** The carbon emissions from the energy consumption and the cement production.

The carbon emissions from energy consumption(10^4^t)	The carbon emissions from cement production(10^4^t)	The total carbon emissions(10^4^t)
962080.4	109661.4	1071741.8

**Fig 2:**
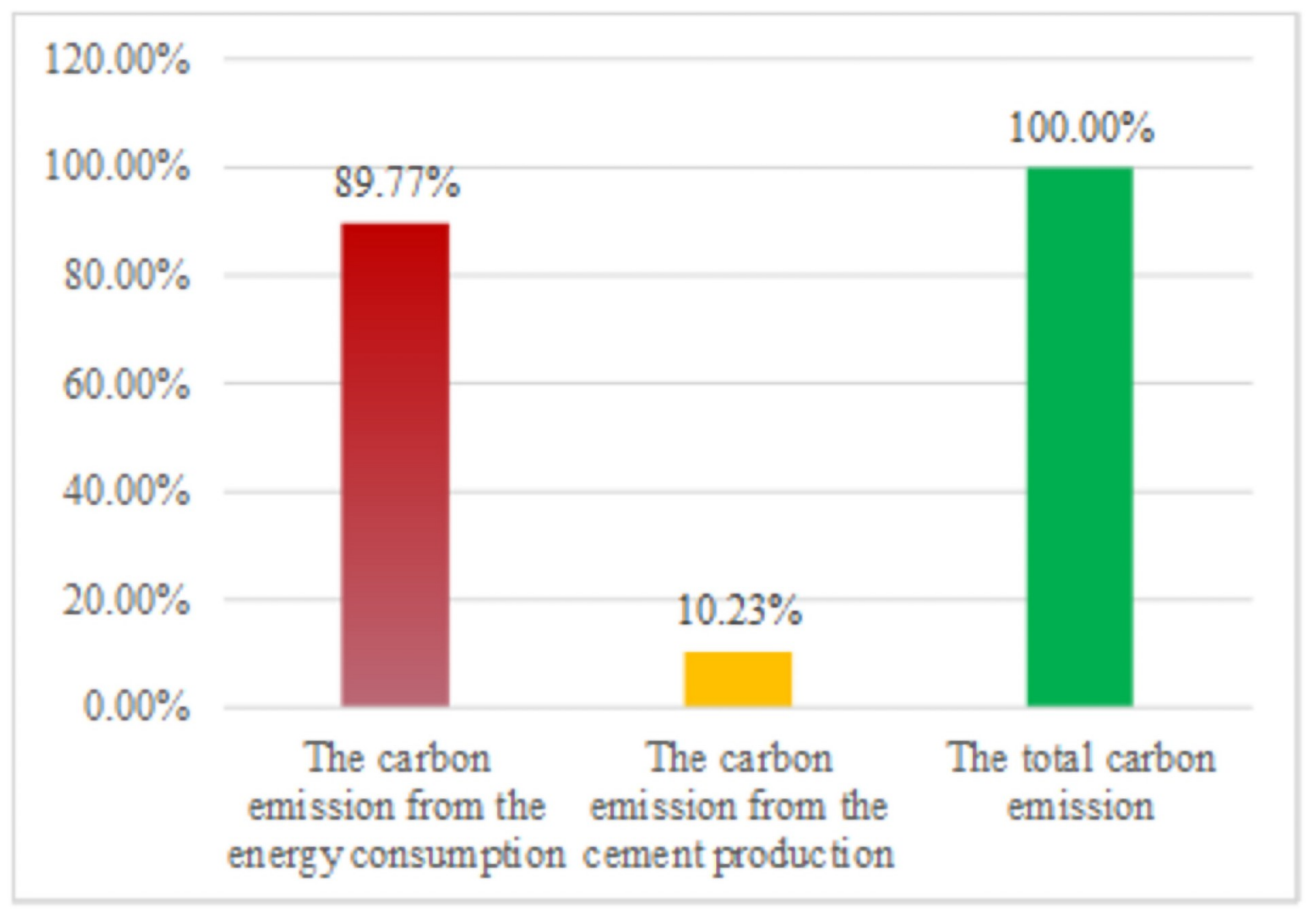
The share of carbon emissions from energy consumption and cement production.

As shown in Fig 2, the carbon emissions from energy consumption accounts for 89.76% of the total carbon emissions, and the carbon emissions from cement production contributes 10.23% of the total amount to China’s carbon emissions (Fig 2). This indicates that in China’s carbon emissions structure, the carbon emissions from cement production have a great impact on China’s carbon emissions structure, and cannot be ignored.

Using the above methods, we can also measure the carbon emissions structure from the energy produced by the four energy supply industries under baseline scenarios. The detailed data are shown in Table 5.

**Table 5:** Carbon emissions from the energy produced by four energy supply industries under baseline scenarios.

Energy industry	Coal mining industry	Petroleum and natural gas exploitation industry	Petroleum, cooking and nuclear fuel products industry	Gas production and supply industry
The CO_2_ emissions(10 kilo-tons)	659360.5	29232.8	227142.4	46344.7

**Fig 3:**
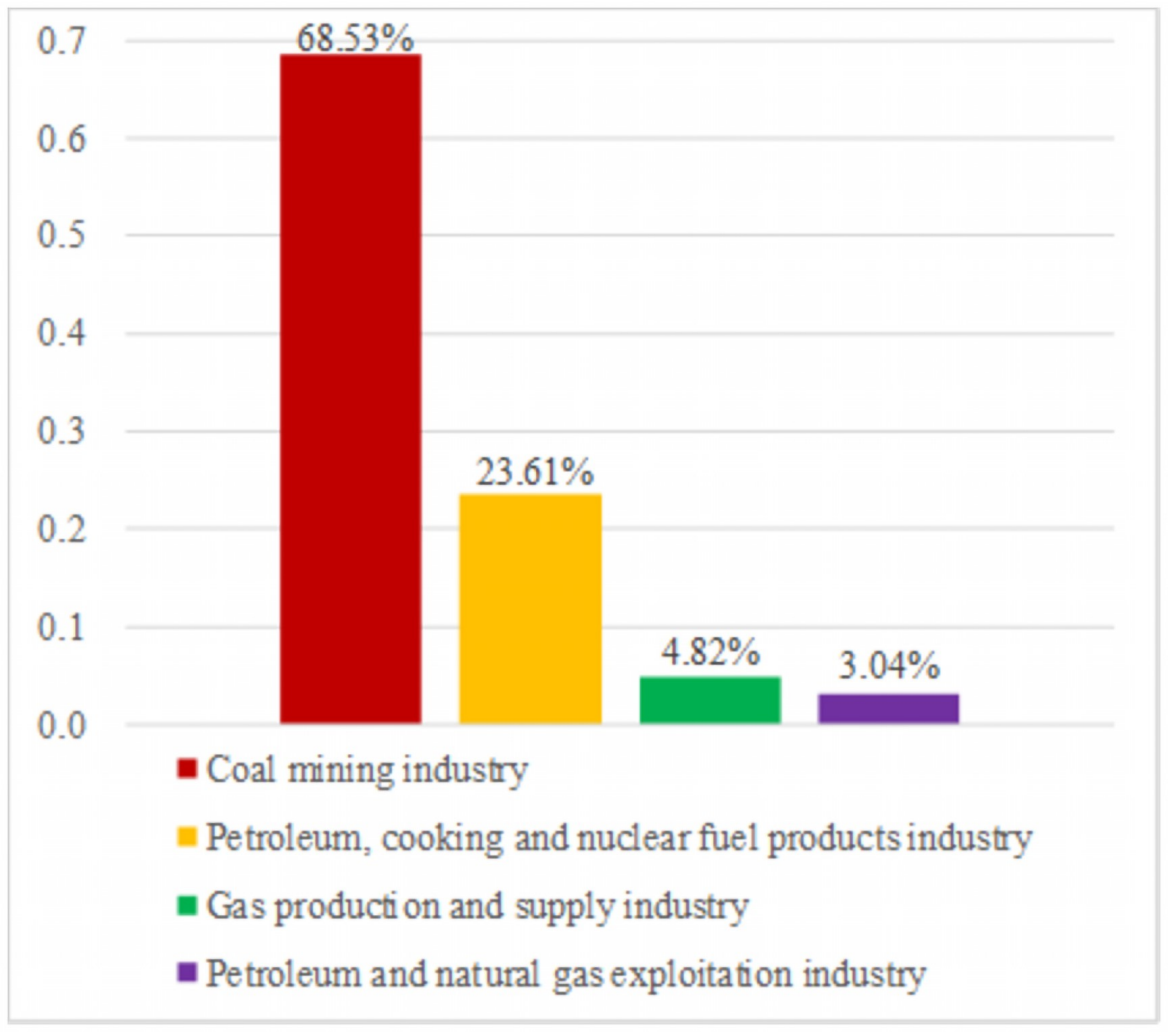
The share of carbon emissions from energy produced by the four energy industries.

According to Table 5, we can obtain the following research result. The carbon emissions from the energy produced by the coal mining industry, petroleum, cooking moreover, nuclear fuel products industry, gas production and supply industry, petroleum and natural gas exploitation industry account for 68.53%, 23.61%, 4.82% and 3.04% of the total carbon emissions from four energy industries (Fig 3). Our data suggest that there is a good scope for optimization of the carbon emissions structure. Thus, we should try to improve the energy production in the gas production and supply industry and petroleum and natural gas exploitation industry, and control the energy production in the coal mining industry.

#### The carbon emissions structure on the demand side of energy

**Fig 4:**
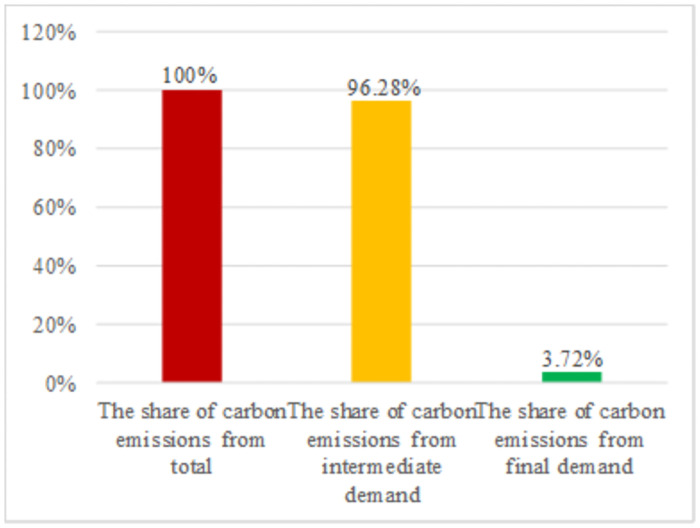
The demand structure of carbon emissions under baseline scenarios.

Using the above data and method, we can measure the carbon emissions structure from the demand side of energy under baseline scenarios. The detailed data is shown in Fig 4.

According to Fig 4, we can obtain two vital sets of information. First, the carbon emissions from the intermediate demand contribute more than 95% of the total amount to China’s carbon emissions. Second, the carbon emissions from final demand contribute less than 5% of the total amount to China’s carbon emissions. Our data suggest that there is a good scope for optimization of China’s carbon emissions structure. Thus, we should try to reduce the proportion of carbon emissions from the intermediate demand and increase the proportion of carbon emissions from the final demand.

### Analysis of carbon emissions and carbon efficiency from different sectors

Using the above model and data, we can obtain the carbon emissions from 42 sectors under the baseline scenario, and we merged 42 sectors into 9 sectors. For details of the merger (the merging process), please see S1 Fig. The carbon emissions and output from the 9 sectors are shown in Table 6.

**Table 6:** The carbon emissions and output from the 9 sectors.

Sectors	The CO2 emissions (10 kilo-tons)	The output (Ten thousand yuan)
Agriculture	8728.593036	894213473.2
Construction	11492.08679	1386125872
Light Industry	26127.28728	2162525697
Service	35980.02854	6612208987
Transportation	65643.69132	619666563.1
Chemical Industry	122667.0174	1210245788
Fossil Energy	136656.5263	378950047.9
Electric Power	283725.161	486933590.6
Heavy Industry	340895.1595	4606416552

**Fig 5:**
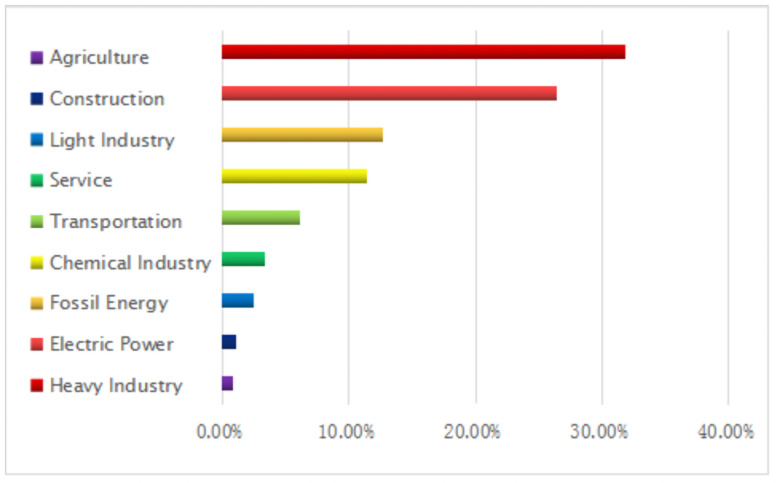
The CO2 emissions from different industries.

**Fig 6:**
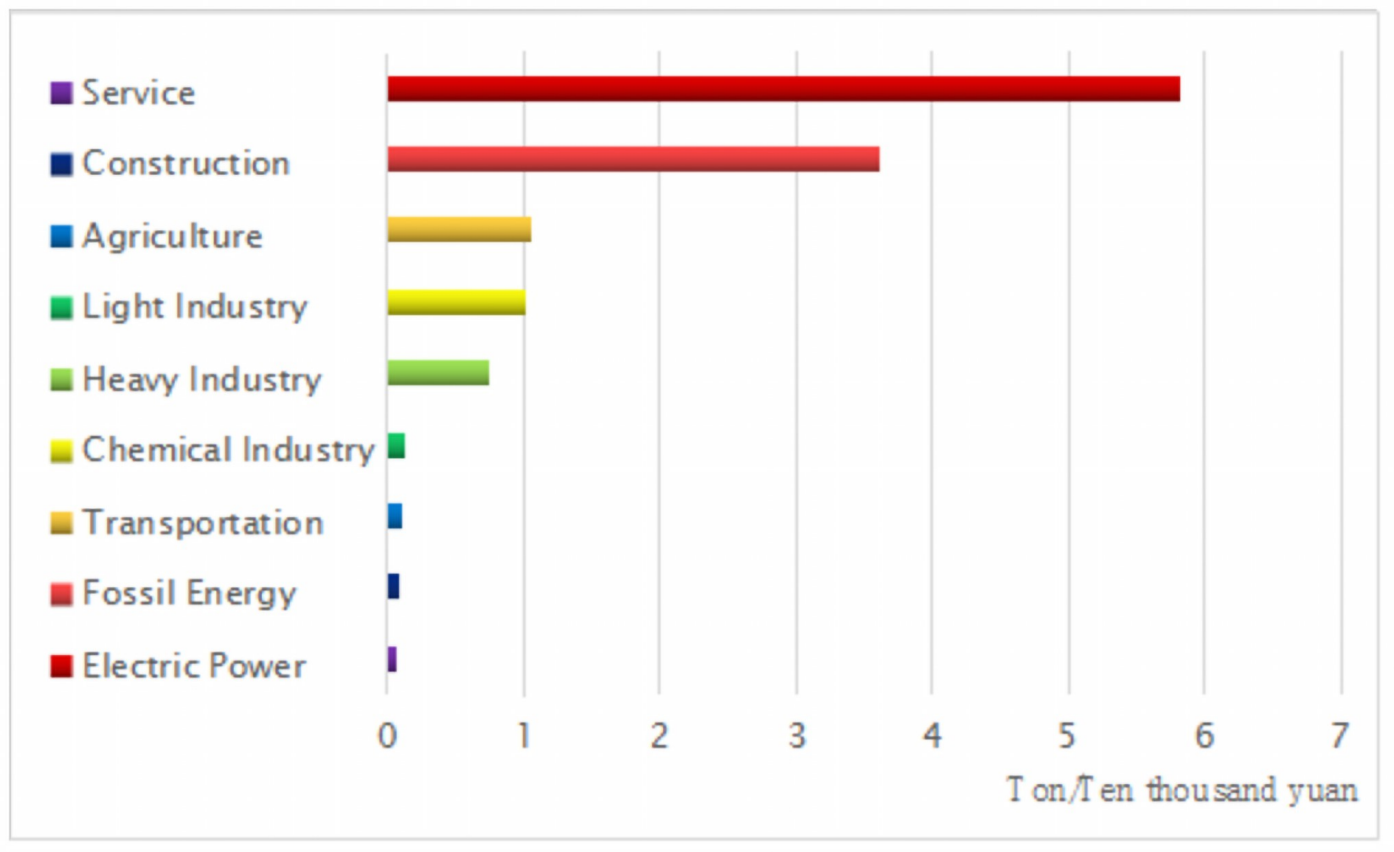
The CO2 emissions efficiency from different industries.

Then we can measure the share of CO2 emissions and carbon emissions efficiency from 9 industries under the baseline scenario, and the result is shown in Figs 5 and 6.

As shown in Fig 5, the heavy industry, electric power, fossil energy, and chemical industry produce large carbon emissions, accounting for 31.81%, 26.47%, 12.75%, 11.45%, and 6.12% of the total emissions. However, the carbon emissions from agriculture, construction, light industry, and service are small, accounting for 0.81%, 1.07%, 2.44% and 3.36% of the total emissions. Comparing the results of this part with the research conclusions of Jiang et al. (2019) and Kang & Yang (2020), we find that the results of this paper are basically consistent with their results, which indirectly strengthens the reliability of the results of this paper, but there is a certain gap in the quantitative results. This is because Jiang et al. (2019) only considered the two factors of energy capital deposit and loan and energy capital export in the analysis carbon emissions from the demand-side of energy, but did not consider the factor of energy conversion, which leads to some errors between the results of this paper and Jiang et al. (2019). Similarly, Kang & Yang (2020) only considered three factors in their paper, but did not consider the influence of carbon emissions from cement production. Therefore, there is a certain error between the research results of this paper and those of Kang & Yang (2020). It should be noted that Liu et al. (2016) pointed out that when calculating carbon emissions from the demand-side of energy, four factors have an impact on carbon emissions calculation. Therefore, considering the four factors, the calculation and analysis of carbon emissions from demand-side is more accurate in theory.

According to the above Fig 6, we can obtain the following research result. The carbon emissions efficiency from electric power, fossil energy, transportation, chemical industry, and heavy industry are low. When they produce 10000 yuan worth of goods, they emit 5.82 tons, 3.60 tons, 1.05 tons, 1.01 tons and 0.74 tons of carbon dioxide. However, the carbon emissions efficiency from service, construction, agriculture, and light industry are high. When they produce 10000 yuan worth of goods, they emit 0.05 tons, 0.08 tons, 0.09 tons and 0.12 tons of carbon dioxide.

Three reasons can explain the above conclusions. Firstly, from the perspective of industrial structure, the heavy industry, electric power, fossil energy, and chemical industry account for a large proportion in China industrial structure, while agriculture and light industry account for a relatively small proportion. Secondly, from the perspective of energy consumption, the power industry and fossil energy sector mainly consume primary energy sources, such as thermal power generation (thermal energy) and coal combustion (fossil fuels). However, agriculture, light industry, and service industry, belong to low-energy industries, mainly consume secondary energy, such as electricity. Thirdly, from the perspective of input factors, agriculture, construction, light industry, and service industry are labor-intensive industries with low energy input, while electric power, chemical industry, heavy industries and fossil energy share large energy inputs.

## Conclusion

In this study, we first used the energy consumption method for measuring the carbon emissions. Then, through an input-output analysis, we investigated the carbon emissions structure on the supply-side and demand-side of energy considering four influencing factors (the energy conversion, energy capital savings and loans, energy exports and cement production carbon emissions). Meanwhile, we analyzed the carbon emissions and carbon emissions efficiency from different industries. The results are as follows. First, if the four influencing factors were not considered, the measurement of carbon emissions from intermediate demand was 12.36% lower than the baseline scenario, 44.91% higher than the final demand and 10.23% lower than the total. Second, from the perspective of energy consumption, the carbon emissions from high-carbon energy, represented by raw coal, accounted for 66.805% of the total emissions from energy sources, while the carbon emissions from low-carbon energy, represented by natural gas, only accounted for 2.485%. The distribution direction of energy use shows that carbon emissions from intermediate use (enterprise production) accounted for more than 95% of total carbon emissions, while carbon emissions from final use (residents and government use) accounted for less than 5%. Third, from the perspective of carbon emissions from the intermediate demand, the heavy industry, electric power, fossil energy, and chemical industry have high carbon emissions and low carbon emission efficiency. However, the agriculture, construction, light industry, and service are the opposite.

Based on the above conclusion, we proposed four suggestions. First, we need to further optimize China’s energy consumption structure, and reduce the consumption proportion of coal as well as other high-carbon energy sources, and increase the proportion of clean energy, e.g., natural gas. This is beneficial for China to achieve green development and pollution control. Second, due to the fundamental importance in improving the measurement accuracy of carbon emissions, the factors of influencing the measurement of carbon emissions should draw more attention in the future. Third, China’s carbon emissions structure has great potential for optimization. We should try to reduce the proportion of carbon emissions from the intermediate demand and increase the proportion of carbon emissions from the final demand. In addition, we should improve the carbon emission efficiency from electric power, fossil energy, transportation, chemical industry, and heavy industry.

## Supporting information

S1 Fig The sector classification. (TIF)
