# Supplementary figures and images for: China’s carbon emissions structure and reduction potential on the supply-side and demand-side of energy: Under the background of four influencing factors
Source: PLoS One. 2021 Aug 6;16(8):e0255387. doi: 10.1371/journal.pone.0255387 (PMC8345844; doi:10.1371/journal.pone.0255387)

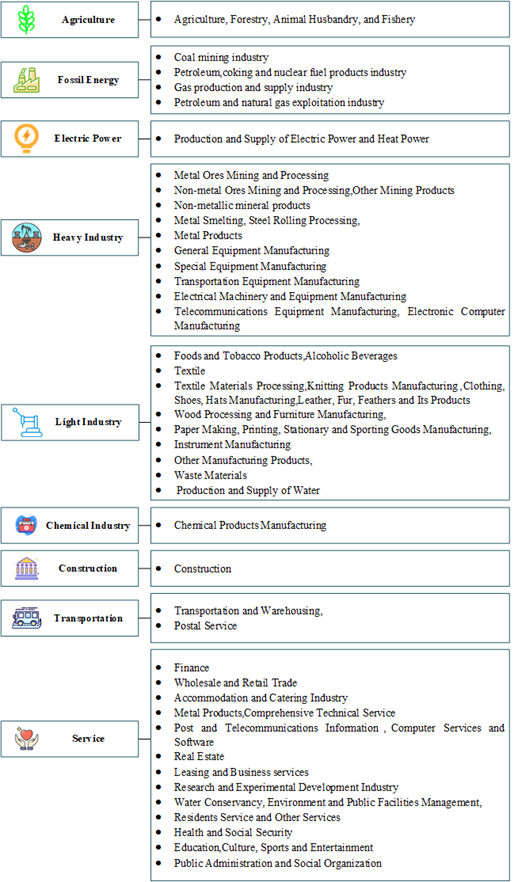

Supplement: S1 Fig — (TIF) [file pone.0255387.s001.tif]
